# Epidemiological Characteristics of Inherited Epidermolysis Bullosa in an Eastern European Population

**DOI:** 10.3390/jcm13133742

**Published:** 2024-06-26

**Authors:** Alina Suru, Sorina Dănescu, Alina Călinescu-Stîncanu, Denis Iorga, Mihai Dascălu, Adrian Baican, George-Sorin Țiplica, Carmen Maria Sălăvăstru

**Affiliations:** 1 Pediatric Dermatology Discipline, Carol Davila University of Medicine and Pharmacy, 8 Eroilor Sanitari Boulevard, 050474 Bucharest, Romania; 2 Pediatric Dermatology Department, Colentina Clinical Hospital, 19-21 Stefan cel Mare Street, 020125 Bucharest, Romania; 3 Dermatology Research Unit, Colentina Clinical Hospital, 19-21 Stefan cel Mare Street, 020125 Bucharest, Romania; 4 Dermatology Discipline, Iuliu Hatieganu University of Medicine and Pharmacy, 8 Victor Babeș Street, 400012 Cluj-Napoca, Romania; 5 Dermatology Department, Saint Spiridon County Emergency Clinical Hospital, No 1 Independenței Boulevard, 700111 Iasi, Romania; 6 Computer Science & Engineering Department, National University of Science and Technology POLITEHNICA Bucharest, 313 Splaiul Independentei, 060042 Bucharest, Romania; 7 Academy of Romanian Scientists, 3 Ilfov Street, 050044 Bucharest, Romania; 8 Second Dermatology Discipline, Carol Davila University of Medicine and Pharmacy, 8 Eroilor Sanitari Boulevard, 050474 Bucharest, Romania; 9 Second Dermatology Department, Colentina Clinical Hospital, 19-21 Stefan cel Mare Street, 020125 Bucharest, Romania

**Keywords:** epidermolysis bullosa, epidemiology, incidence, prevalence

## Abstract

**Background/Objectives**: Epidermolysis bullosa (EB) is a hereditary condition characterized by skin and mucosal fragility, with various degrees of severity. This study’s objectives are to obtain updated epidemiological data that will help identify the specific types and subtypes of EB, determine the case distribution in Romania, and establish the incidence and prevalence of the condition. **Methods**: This population-based observational study included Romanian patients and collected data from 2012 to 2024. The following information was recorded: date of birth, status (deceased or alive), date of death (if applicable/available), sex, county, and city of residence, EB type and subtype if available, diagnosis (clinical and/or immunofluorescence mapping, transmission electron microscopy, genetic molecular analysis), affected genes, inheritance, and affected family members. **Results**: The study included a total of 152 patients. The point prevalence (the proportion of the population with a condition at a specific point in time) and the incidence of EB in Romania were 6.77 per million population and 24.23 per million live births, respectively. EB simplex (EBS), junctional EB (JEB), dystrophic EB (DEB), Kindler EB (KEB), and not otherwise specified EB, as well as EB (NOS), were the main types of the condition identified in 21%, 3%, 63%, 2%, and 11% of the total cases. The point prevalence and incidence for the same time intervals were 1.58 and 5.28 in EBS, 0.10 and 1.76 in JEB, 4.72 and 12.34 in DEB, 0.16 and 0 in KEB, and 0.21 and 4.85 in EB (NOS). **Conclusions**: The study provides updated epidemiological data for Romania and underlines the necessity for accurate diagnosis, facilitated by access to genetic molecular testing and better reporting systems.

## 1. Introduction

Epidermolysis bullosa (EB) comprises an array of hereditary disorders characterized by the fragility of the skin. These disorders develop due to genetic mutations in the proteins responsible for cellular adhesion. Considering the most recent consensus reclassification, EB is divided into four major categories: (a) intraepidermal (EB simplex), (b) junctional (junctional EB), (c) dermal (dystrophic EB), and (d) mixed (Kindler EB), depending on the location of the blister relative to the dermal-epidermal junction. Furthermore, EB types are categorized, based on their clinical severity, as severe, intermediate, and localized [1].

EB is associated with a vast spectrum of clinical manifestations, assembled in different clinical forms [2]. Mild subtypes of EB are characterized by minimal mucosal and visceral involvement, allowing individuals to lead a relatively normal life. However, misdiagnosis and underreporting often occur in these milder forms of EB. On the other hand, severe recessive variants of EB are highly debilitating, causing significant physical disfigurement and negatively impacting both quality of life and life expectancy [3]. The disease’s onset is usually immediately after birth and can affect both the skin and the mucosa.

In Europe, various studies report the prevalence and incidence of EB (see Table 1). Prevalence reports vary; 20 cases per million individuals are reported in Slovenia [4] and a much higher number, 54.03 cases per million individuals, in Germany [5]. The incidence ranges from 1.4 per million births in Northern Ireland [6] to 67.8 in England and Wales [7]. In the United States, data published in 2016 indicated a prevalence of 11.1 cases per million individuals and an incidence of 19.57 cases per million births [8]. In Europe, accurate epidemiological data is reported from countries with long-established EB registers and well characterized patient cohorts, as in the Netherlands [9] or England and Wales [7].

The most recent data regarding EB epidemiology in Romania comes from 2015, when it was estimated that the condition’s prevalence was 4.42 cases per 1 million people, and the incidence was 25 cases per 1 million newborns [10].

The objectives of this population-based, observational and retrospective study were to obtain updated epidemiological data, identify the specific types and subtypes of EB, determine the case distribution in Romania, and establish the incidence and prevalence of the condition in Romania. In this sense, the following section focuses on describing the materials and methods used to achieve the objectives of the study.

**Table 1:** Epidemiology of EB in various countries in Europe, North America, Australia, and New Zealand.

Country	EB Prevalence (All Types) Per Million Residents	EB Incidence (All Types) Per Million Births
Romania (this study population sample)	6.77	24.23
Slovenia [4]	20	NA
Germany [5]	54.3	45
United Kingdom England and Wales [7] Scotland [11] Northern Ireland [6]	34.8 49 32	67.8 NA 1.4
France [12]	12.7	NA
Italy 2005 [13]	10.1	0.1
1991–2007 [8]	15.4	NA
The Netherlands [9]	22.4	41.3
Hungary [14]	≈15	NA
Norway [15]	54	NA
Russia [16]	3.6–3.9	0.22–0.33
USA [8]	11.1	19.57
Australia [17]	10.3	NA
New Zealand [18]	19.5	NA

## 2. Materials and Methods

### 2.1. Data Collection

In Romania, five University hospitals are affiliated with the National Program for the Treatment of Rare Skin Diseases—epidermolysis bullosa. Patients with EB are usually registered with one of these centers in Bucharest, Cluj-Napoca, Iasi, Timisoara, or Târgu-Mures. These institutions provide ongoing care to affected patients. 

All EB centers, and the Romanian association of EB patients, MiniDebra, were invited to participate to this study. Approval was granted by the Ethics Committee of Colentina Clinical Hospital (Date 1 April 2024/No 8). We collected data from 1 January 2012 to 1 May 2024.

We aimed to collect the following data: date of birth, status (deceased or alive), date of death (if applicable/available), sex, county, and city of residence, EB type and subtype if available, diagnosis (clinical and/or immunofluorescence mapping, transmission electron microscopy, genetic molecular analysis), affected genes, inheritance, and affected family members. 

We excluded patients with acquired epidermolysis bullosa and other skin fragility syndromes. We included patients with confirmed diagnoses, patients who received a diagnosis based on the suggestive clinical aspect, as well as those who underwent genetic molecular analysis, but the results were inconclusive, pending a definitive diagnosis. 

Romanian patients with epidermolysis bullosa may address other EB centers in Europe (e.g., Austria, United Kingdom, Italy) for a second opinion regarding the diagnosis, for diagnostic testing, for consults regarding the complications of the severe forms of EB, etc. These patients were included in this analysis, as they are registered with the Romanian National Health Care system.

### 2.2. Duplicate and Missing Data

To avoid data duplication, the collected data was cross-referenced, and patients who had a double registration, both with an EB center and with MiniDebra, or in two EB centers, were retained at a single EB center.

In terms of missing data, we received results only from the centers in Bucharest, Cluj-Napoca, and Iasi, as well as from the patient association MiniDebra. The lack of data from the other two centers could not be addressed.

While considering the data received from MiniDebra, 46 cases of living patients that were not duplicates with other centers lacked essential details, such as date of birth, county, type of diagnosis, or other information. Efforts were made to contact these patients by telephone to obtain the missing data. However, relevant information was retrieved from only 20 of the 46 patients due to various reasons, including refusal to participate, insufficient information provided, no response, or the lack of a confirmed diagnosis.

The date of birth was missing for two cases from the Bucharest and Cluj centers. For these, we computed the year of birth using data on age and assumed the 1st of January as the day and month of birth. Additionally, three cases were lost to follow-up with no information on the exact date of censoring. Here, we assumed that these three cases were alive at the time of data collection, as they were known to be affected by milder phenotypes, so this assumption was reasonable.

Regarding deceased patients, the specific month and day of death were missing for 15 cases (though the year was available). For 2 cases, the month of death was inferred using the date of birth and age, and the day of death was assumed to be the first day of that month. For the remaining 13 death cases, only the year of birth, the year of death, and the age at death were available; as such, the 1st of January was assumed to be the day and month of death. The month and day of birth in these instances were computed by subtracting the assumed date of death from their age.

Taken together, the procedures of assuming specific days and months of birth or death had no impact on the calculation of incidence measurements and a minimal impact on computing prevalence and mortality metrics.

### 2.3. Diagnosis

The diagnosis of EB was determined through an evaluation of clinical characteristics and paraclinical tests, such as immunofluorescence antigen mapping (IFM), transmission electron microscopy (TEM), and genetic molecular testing, depending on availability. 

Clinical diagnosis was based on the recognition of the clinical signs associated with the condition: skin and mucosal fragility, nail dystrophy, mitten deformities of the hands and feet, and oral involvement, all easily recognized in older children and adults [2,3]. Immunofluorescence mapping is a fast technique useful for identifying different severe subtypes of EB in newborns by detecting the lack of immunoreactivity of specific proteins [19]. Transmission electron microscopy enables the precise determination of the blister level within the skin. A thorough understanding of epithelial adhesion structures in both healthy and EB skin is essential for interpreting TEM. It is a valuable method, but it is expensive, time-consuming, and requires high expertise [19,20]. Molecular genetic testing should follow these diagnostic techniques [5].

The most recent consensus report [1] guided the classification of EB, but not all reported cases had a complete diagnosis that included the type and subtype of the condition.

### 2.4. Data Analysis

Prevalences were calculated per million population. EB point prevalence was calculated for the entire study population and for each EB [total number of EB patients alive at a time point (31 December 2023)/total population at that time point (31 December 2023) × 1,000,000]. Incidences were calculated per million live births [total number of new patients with EB born (1 January 2012 to 31 December 2022)/total number of live births (2012–2022) × 1,000,000]. We obtained data on Romania’s demographic situation from the National Institute of Statistics (http://statistici.insse.ro:8077/tempo-online/#/pages/tables/insse-table, accessed on 15 May 2024).

The epidemiological data were analyzed using cross tables and data visualization methods in Python (version 3.10.12), R (version 4.2.3), and Excel (version 16.86). Python and Excel were used to compute the prevalence and incidence measurements. Two independent researchers computed the prevalence and incidence measurements to ensure reliability. R software and Excel were used to create visualizations. R software was used to compute the Kaplan–Meier estimates. The “survival” and “survminer” packages were used to calculate survival curves for the main types of EB and generate the Kaplan–Meier plot.

## 3. Results

We have received data on 241 entries: 125 cases from the EB centers (58 cases from Bucharest, 38 patients from Cluj-Napoca, and 29 patients from Iasi), and 116 cases from the patient’s association MiniDebra. After cross-referencing and the removal of duplicates, we considered 152 cases, 119 cases from the EB centers (56 cases from Bucharest, 36 patients from Cluj-Napoca, and 27 patients from Iasi), and 33 cases from MiniDebra (20 cases of patients that are alive and 13 of deceased patients; for this last category of deceased patients, the date of death and age at death are known, but the type of EB is not).

### 3.1. Diagnosis

We classified each diagnosis into one of the subsequent categories: EBS with localized EBS, intermediate EBS, severe EBS, EBS suspected, non-specified or congenital that were grouped as not otherwise specified (NOS) EBS; JEB (Junctional EB) with severe JEB and not otherwise specified JEB; DEB with DDEB (dominant dystrophic EB), RDEB (recessive dystrophic EB), DEB not otherwise specified (NOS); KEB (Kindler EB), and congenital and not otherwise specified EB (NOS).

### 3.2. Epidermolysis Bullosa Types and Subtypes

**Figure 1:**
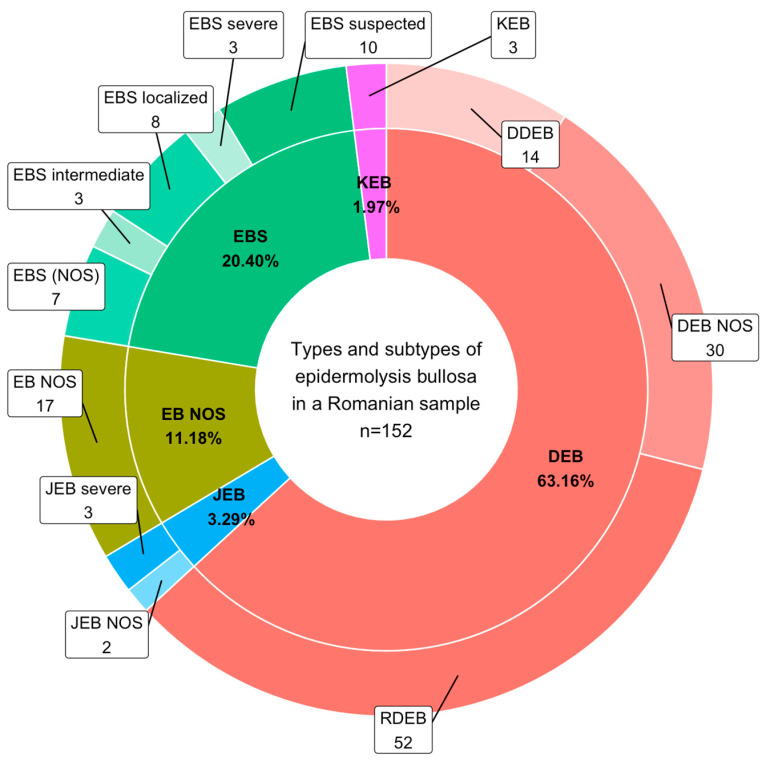
Main epidermolysis bullosa types and subtypes (EBS, EB simplex; JEB, junctional EB; DEB, dystrophic EB; EB (NOS), EB not otherwise specified).

Figure 1 displays the main types of EB reported, which include 31 cases of EBS (20.40%), 5 cases of JEB (3.29%), 96 cases of DEB (63.16%), 3 cases of KEB (1.97%), and 17 cases of EBS (NOS) (11.18%). We reported a total of 31 cases of EBS, including 8 (26%) of localized EBS, 3 of intermediate EBS (10%), and 3 of severe EBS (10%), 7 (22%) of EBS (NOS), and 10 (32%) cases of suspected EBS. We identified 5 cases of JEB, of which 3 (60%) were severe and 2 (40%) were not otherwise specified (NOS). The most reported cases were DEB, with 96 reported cases. Out of these, 52 cases (54.17%) were RDEB, 14 cases (14.58%) were DDEB, and 30 cases (31.25%) were other DEB cases (7 DEB severe and 23 not otherwise specified). Figure 1 illustrates the DEB subtypes. There were also three (1.97%) cases of KEB reported, while 17 (11.18%) cases of EB were not otherwise specified.

### 3.3. Type of Diagnosis 

**Figure 2:**
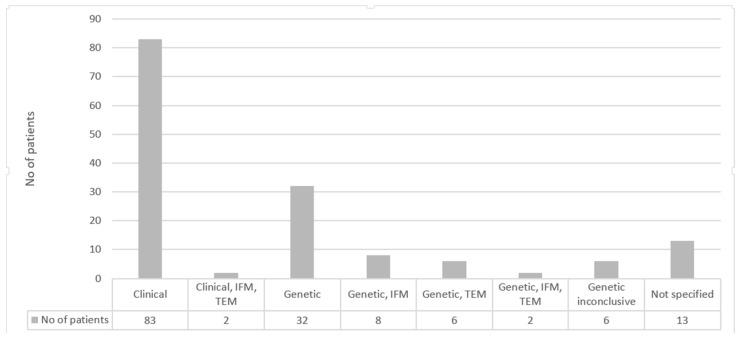
Type of diagnosis in epidermolysis bullosa patients (IFM, immunofluorescence mapping; TEM, transmission electron microscopy).

All patients were evaluated clinically. As discussed above, the complete diagnosis should employ IFM and/or TEM (first step), and it should be followed by genetic testing. The majority of patients, 83 (54.60%), received the diagnosis based only on clinical observations, with only 2 (1.3%) having IFM and TEM that supported the clinical findings; 48 (31.5%) had genetic molecular testing that established the diagnosis. Of these 48 patients, 8 had IFM and genetic molecular testing, 6 had TEM and genetic molecular testing, and only 2 received a diagnosis based on the three diagnostic methods (genetic, IFM, and TEM). For 6 (3.94%) patients, the genetic molecular testing yielded inconclusive results, whereas for 13 (8.5%) patients, we received no diagnosis information (see Figure 2).

Regarding the inheritance, 58 cases were reported as having autosomal recessive inheritance, 29 had autosomal dominant inheritance, 3 cases had de novo mutations, while the inheritance was not known or was not specified for 62 cases. For EBS patients who had genetic molecular testing, variants in *KRT 5* were identified in 2 cases, and for *KRT 14*, they were identified in 5 cases. We identified variants in *COL7A1* in 35 DEB cases, and in *FERMT1* in 3 KEB cases. Two patients had variants in *LAMA3* and *LAMB3*, respectively. 

### 3.4. Distribution of Cases According to Age and Sex

**Figure 3:**
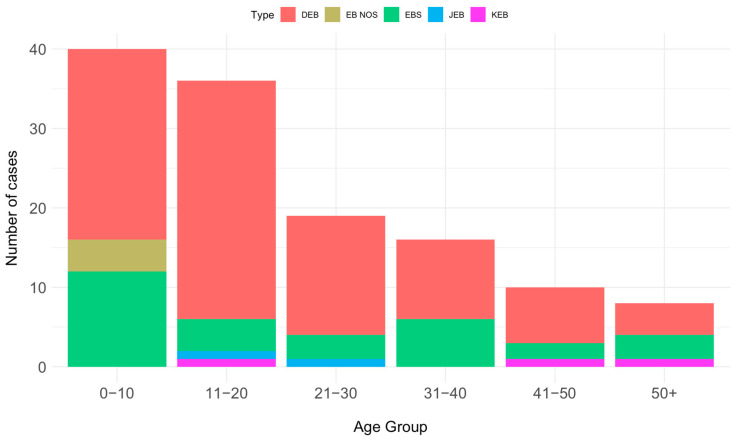
Distribution of cases according to age split per main types of epidermolysis bullosa (DEB, dystrophic EB, EB (NOS), EB not otherwise specified; EBS, EB simplex; JEB, junctional EB; KEB, Kindler EB).

The distribution according to patient age showed a decreasing pattern. The majority of patients (40) fell into the 0–10 year age group, followed by 36 patients in the 11–20 year age group, 19 patients in the 21–30 year age group, 16 in the 31–40 age group, and 10 patients in the 41–50 year age group. Only eight patients were over 51 years old. Moreover, there was an even distribution of cases, with a slight female preponderance compared to males, specifically 67 (51.94%) versus 62 (48.06%). Figure 3 depicts the distribution according to the age of the main EB types, for alive patients.

### 3.5. Geographic Distribution 

**Figure 4:**
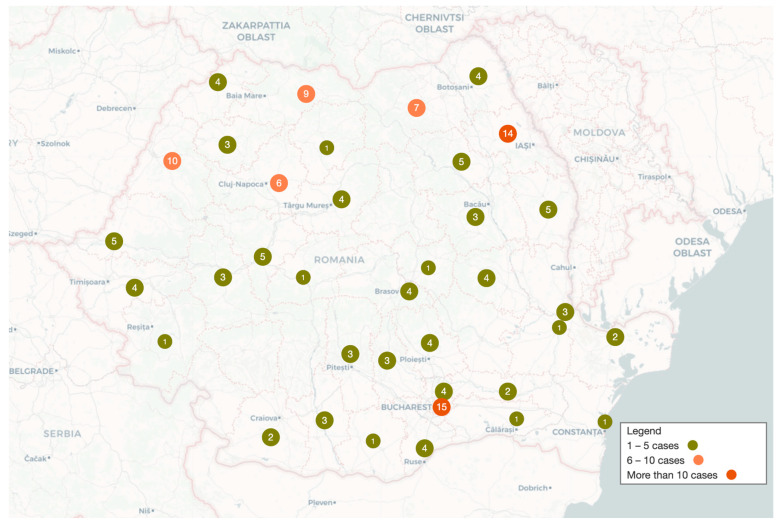
Geographic distribution of epidermolysis bullosa patients in Romania with county distribution of cases.

Figure 4 shows the distribution of cases by the patient’s home county. As expected, most cases are clustered around the three largest centers that provided data in the southeast, northwest, and northeast of the country, respectively, around Bucharest, Cluj-Napoca, and Iasi.

### 3.6. Prevalence and Incidence

Table 2 and Appendix A detail the prevalence and incidence values for the main types and EB subtypes. The point prevalence of epidermolysis bullosa was calculated referencing the date of 31 December 2023, and per million of the population, according to the National Institute of Statistics (19,054,548 total population, 31 December 2023, http://statistici.insse.ro:8077/tempo-online/#/pages/tables/insse-table).

The most prevalent type of EB was DEB at 4.72 per million, followed by EBS at 1.58 per million, not specified EB (EB NOS) at 0.21, KEB at 0.16 per million, and JEB at 0.10 per million.

To calculate the incidence of the disease, we used the information on patients with EB born between 1 January 2012, and 31 December 2022, as well as data regarding the total live births according to the National Institute of Statistics in the same period (2,269,591 live births, 1 January 2012, and 31 December 2022 http://statistici.insse.ro:8077/tempo-online/#/pages/tables/insse-table).

Over this period, 55 live-born babies with EB were registered, and the incidence per million live births was 24.23 for EB (all types), 5.28 for EBS, 1.76 for JEB, 12.34 for DEB, and 4.85 for EB (NOS).

### 3.7. Mortality

Since 2012, we have recorded 23 deaths: one EBS case, three JEB cases, six DEB cases, and 13 unspecified cases. The mean age at death was 13 years; seven patients were younger than one year, and the oldest patient was 43. For the three severe JEB cases, the mean age at death was 9.6 months; for DEB, it was 23 years; and for the unspecified cases, it was 11.89 years, suggesting that most of the cases had a severe type of EB with an unfavorable prognosis. 

**Figure 5:**
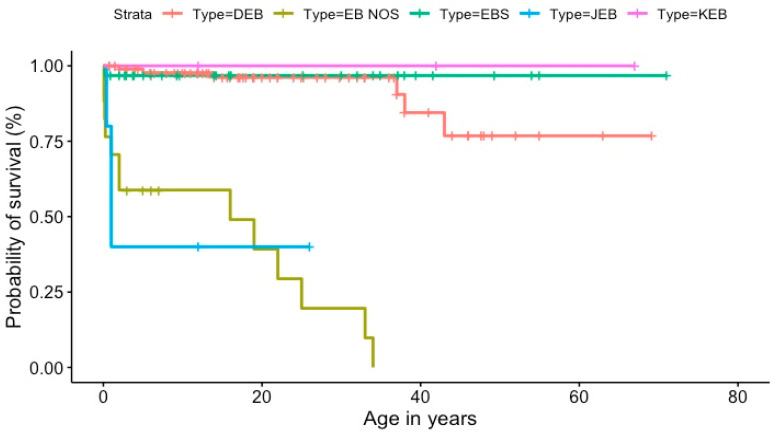
Kaplan–Meier graph illustrating survival rates for the EB patients registered since 2012 (DEB, dystrophic EB, EB (NOS), EB not otherwise specified; EBS, EB simplex; JEB, junctional EB; KEB, Kindler EB).

We calculated the probability of survival for the different EB types based on the reported data. The Kaplan–Meier plot shows that JEB patients and the non-specified cases had the lowest survival probability, around 40% at the age of one for JEB; the survival probability was around 20% at the age of 30 for EB (NOS) (see Figure 5).

## 4. Discussion

Healthcare systems may benefit from identifying the number and needs of patients with rare skin conditions, including EB. These epidemiological data are critical for therapeutic trials and help to understand the widespread need for specialized medical treatment among EB patients [21]. Families in Romania face a significant financial burden due to gaps in public health coverage for EB-related care. Enhancing the support systems and healthcare infrastructure is essential for better EB management.

Romania benefits from EB expertise centers, but there are limitations in terms of availability and reimbursement for diagnostic possibilities. Like other European countries, Romania has seen an increase in patient recruitment in the last decade [12], highlighting the improvements in patient care and the establishment of expert centers following the government-financed rare disease program. Epidermolysis bullosa was included in the National Program for the Treatment of Rare diseases in 2012. The program aims to help EB patients receive some of the medical supplies needed for daily care, such as dressings, gauzes and topical medication”.

Regarding the main types of EB, 17 cases (11.2%) were not specified (see Figure 1). For each type of EB, there were situations where no accurate information existed to facilitate subclassification. This was particularly true for EBS, with 17 cases (54%) reported as not otherwise specified or suspected, and for DEB, with 30 cases (31%) not otherwise specified. Germany [5] and Australia [17] have reported similar results regarding EBS, indicating a high percentage of unclassified cases.

Regarding the number of cases of each subtype, the small number of EBS cases (21%) compared to DEB cases (63%) can be explained by the fact that patients with mild symptoms that do not require a lot of medical care are not registered with an EB center or the patient association and remain undiagnosed. Furthermore, the rarity of EB may contribute to a lack of awareness regarding the disorder, which could result in overlooked diagnoses. France also reported a higher prevalence of DEB over EBS [12]. 

Genetic molecular testing diagnosed only 54 (35.44%) of the patients, confirming the diagnosis for 48 (31.57%) of cases, while 83 (54.6%) received a clinical diagnosis. The percentage of diagnoses by IFM and/or molecular testing is lower than for other centers in Europe (66.2% in Germany [5], 90% in the UK [7], and 90.5% in the Netherlands [9]). Due to the limited availability and reimbursement of diagnostic tests in Romania, a significant portion of EB diagnoses are based on clinical features. This impacts the precision of subclassifications. 

Compared to other countries, especially in Europe [5,7,9], but also in the US [8], we noted higher reported incidence and prevalence values; these countries have robust healthcare systems and unified services that provide diagnosis and care for EB patients (see Table 3).

Regarding the incidence and prevalence of EB, our reported values are similar to those published in 2015 in Romania: 24.23 per million live births and 6.77 per million population, versus 25 per million live births and 4.42 per million population [10]. The EB expert centers have been actively retaining patients in the last decade, and the healthcare community has begun to refer more patients to these centers, which could explain the difference in prevalence numbers. Furthermore, the EB patient community and patient association were more active in registering new cases.

In 2022, in the first study to report EB prevalence in France [12], the authors underlined the importance of having precise diagnosis of EB cases, well-developed reporting systems, and consistent epidemiological analyses that adhere to defined protocols. These measures were considered essential for enabling meaningful comparisons across different countries.

### Study Strengths and Limitations

Strengths: Cases were reported from all regions of the country, from the three main EB centers in Romania, but also from MiniDebra, the national organization of patients suffering from EB.

Limitations: The under-recruitment of patients with less severe types of EB, especially those with EBS, is an acknowledged problem in all epidemiological studies of EB [17]. Missing data from two centers (Timisoara and Târgu-Mures) could have biased the results. Nonetheless, the use of data from MiniDebra partially addressed this limitation. Still, 24 cases from MiniDebra were excluded as they could not be validated. These issues may explain the lack of any cases in five counties (Mehedinti, Gorj, Valcea, Buzau, Harghita). Considering these aspects, it is possible for the incidence and prevalence to be underestimated.

Furthermore, several assumptions had to be made in order to address the missing data regarding the specific day and month of death, birth, or censoring for 20 cases. Follow-up analyses revealed that this data imputation approach had no impact on incidence and minimal impact on prevalence and mortality estimates.

Calculating the point prevalence and death rates for EB and its variants in Romania is more problematic, due to the absence of a national health record that would provide similar data available in other countries, such as the Netherlands [9] or the UK [7]. Regarding mortality, the lack of specificity in the majority of reported deaths regarding the type of EB they experienced renders the mortality data incomplete, making it impossible to compare with the figures found in the literature.

## 5. Conclusions

This study highlights the current epidemiological landscape of epidermolysis bullosa in Romania, illustrating the distribution, types, diagnostic methods, and prevalence/incidence rates of this rare skin condition. The findings indicate a prevalence of 6.77 cases per million population and an incidence of 24.23 cases per million live births, numbers that align closely with previous reports from 2015. Dystrophic EB emerged as the most prevalent type, reflecting a significant challenge in terms of medical and financial burden. Despite the existence of dedicated EB centers in Romania and improvements in patient diagnosis and care over the last 12 years, access to diagnostic capabilities is still limited, mostly due to financial constraints; as such, there is a high reliance on clinical observations over genetic and other advanced testing methods. This limitation hampers precise subclassification and underlines the need for improved diagnostic infrastructure and support systems.

In conclusion, the epidemiological insights provided by this study are crucial for future healthcare policies and resource allocation for the welfare of EB patients. There is a pressing need for increased awareness, better access to diagnostic resources, and comprehensive care programs to address the complex needs of these patients and their families and improve their quality of life. Future efforts should focus on increasing access to modern diagnostic resources and ensuring equitable access to care across the country.

## Figures and Tables

**Table 2:** Point prevalence for EB types and subtypes on 31 December 2023 and incidence between 2012 and 2022 per million of the population using data from the National Statistics Institute (http://statistici.insse.ro:8077/tempo-online/#/pages/tables/insse-table) (EBS, EB simplex; JEB, junctional EB; DEB, dystrophic EB, KEB, Kindler EB, EB (NOS) EB not otherwise specified).

EB Type	EB Subtype	Point Prevalence on 31 December 2023	Incidence between 2012 and 2022
EBS	All EBS	1.58	5.28
	EBS suspected	0.53	2.20
	EBS (NOS)	0.37	0.44
	Localized	0.42	1.32
	Intermediate	0.16	0.88
	Severe	0.10	0.44
JEB	All JEB	0.10	1.76
	JEB (NOS)	0.10	0.44
	JEB severe	0	1.32
DEB	All DEB	4.72	12.34
	DEB (NOS)	1.57	4.85
	RDEB	2.41	6.17
	DDEB	0.74	1.32
KEB		0.16	0
EB (NOS)		0.21	4.85
ALL EB		6.77	24.23

**Table 3:** Comparison of prevalence and incidence at the international level.

EB Type	Romania		The Netherlands [9]		England and Wales [7]		Germany [5]		US [8]	
	Prevalence per Million Population	Incidence per Million Live Births	Prevalence per Million Population	Incidence per Million Live Births	Prevalence per Million Population	Incidence per Million Live Births	Prevalence per Million Population	Incidence per Million Live Births	Prevalence per Million Population	Incidence per Million Live Births
ALL EB	6.77	24.23	22.4	41.3	34.8	67.8	54.02	45.09	11.1	19.57
EBS	1.57	5.28	11.9	17.5	17	32.5	28.44	14.93	6.0	7.87
JEB	0.10	1.76	2.1	9.3	1.0	8.9	2.44	14.23	0.49	2.68
DEB	4.72	12.33	8.3	14.1	10.7	26.1	12.16	15.58	3.8	-

## Data Availability

Partial data is included in the Appendix A. Further inquiries can be addressed to the corresponding author.

## Appendix A

**Table A1:** Epidermolysis bullosa types and subtypes, cases (numeric and percentages), cases alive on 31 December 2023, point prevalence for EB types and subtypes, number of patients born between 1 January 2012 and 31 December 2022 and calculated incidence for each EB type.

EB Type	EB Subtype	Cases All	Alive 31 December 2023	Point Prevalence	Born 1 January 2012–31 December 2022	Incidence
EBS	All EBS	31 (21%)	30	1.58	12	5.28
	EBS suspected	10 (6.77%)	10	0.53	5	2.20
	EBS (NOS)	7 (4.74%)	7	0.37	1	0.44
	Localized	8 (5.41%)	8	0.42	3	1.32
	Intermediate	3 (2.03%)	3	0.16	2	0.88
	Severe	3 (2.03%)	2	0.10	1	0.44
JEB	All JEB	5 (3%)	2	0.10	4	1.76
	JEB (NOS)	2 (1.2%)	2	0.10	1	0.44
	JEB severe	3 (1.8%)	0	0	3	1.32
DEB	All DEB	96 (63%)	90	4.72	28	12.34
	DEB (NOS)	30 (19.68%)	30	1.57	11	4.85
	RDEB	52 (34.12%)	46	2.41	14	6.17
	DDEB	14 (9.18%)	14	0.74	3	1.32
KEB		3 (2%)	3	0.16	0	0
EB (NOS)		17 (11%)	4	0.21	11	4.85
ALL EB		152 (100%)	129	6.77	55	24.23
Romanian population 31 December 2023		19,054,548				
Live births 2012–2022		2,269,591				
